# Lower reward sensitivity in frontostriatal stroke: Influence of depression and resting-state functional connectivity

**DOI:** 10.3758/s13415-025-01318-9

**Published:** 2025-06-06

**Authors:** Ana Sánchez-Kuhn, Pilar Fernández-Martín, Rocío Rodríguez-Herrera, José García-Pinteño, José Juan León, Miguel Soto-Ontoso, Laura Amaya-Pascasio, María Alonso de Leciñana, Patricia Martínez-Sánchez, Pilar Flores

**Affiliations:** 1 Facultad de Ciencias de La Salud, Universidad Internacional de La Rioja, La Rioja, Spain; 2 Faculty of Psychology, Department of Psychology, CTS-280 Clinical and Experimental Neuroscience Research Group and Research Center CiBiS, University of Almeria, La Cañada de San Urbano, 04120 Almeria, Spain; 3 Mental Health and Neurology Units, Torrecardenas University Hospital, 04009 Almeria, Spain; 4 Department of Neurology, Universitary Hospital La Paz, 28046 Madrid, Spain

**Keywords:** Stroke, Reward sensitivity, Probabilistic reversal learning task, Depression, Functional connectivity, Functional near-infrared spectroscopy

## Abstract

Stroke patients have shown low reward sensitivity, which is a transdiagnostic dimension that defines the extent to which a person actively pursues rewarding stimuli. Low reward sensitivity has been related to depression and dysregulation of the frontostriatal network. To date, studies have addressed this dimension in heterogenic stroke lesions and the underlying mechanisms of frontostriatal stroke patients are still unknown. This study included 54 participants (32 chronic frontostriatal stroke patients and 22 healthy controls). Reward sensitivity was assessed using the probabilistic reversal learning task. Depressive symptoms were measured with the Adult Self-Report, and resting-state functional connectivity (rsFC) was examined using functional near-infrared spectroscopy (fNIRS) in prefrontal, motor, and parietal cortices. Group differences and predictors of reward sensitivity were analyzed using Bayesian ANCOVA and multiple regression models. Stroke patients displayed lower reward sensitivity, higher depressive problems, and lower resting-state functional connectivity between the right orbitrofrontal cortex and the left dorsolateral prefrontal cortex, the right orbitrofrontal cortex and the right dorsolateral prefrontal, and the right dorsolateral prefrontal cortex and right premotor cortex and supplementary motor area. In stroke patients, lower reward sensitivity was predicted by higher depressive problems and lower resting-state functional connectivity between the right dorsolateral prefrontal cortex and the right premotor cortex and the right supplementary motor area. This work showed the relevance of reward sensitivity in frontostriatal post-stroke patients and its relationship with depression, and supports the resting-state functional connectivity measurement for characterizing abnormalities in connectivity in stroke patients.

**Supplementary Information:**

The online version contains supplementary material available at 10.3758/s13415-025-01318-9.

## Introduction

Stroke is the first cause of disability worldwide and the second leading cause of death (Feigin et al., 2018). Lanas and Seron (2021) found that 6 months after stroke 55.9% of patients had died or were disabled, and 61% after 12 months (Lanas & Seron, 2021). The possibility of suffering a stroke has increased by 50% over the last 17 years. Currently, one person in four is estimated to develop a stroke in their lifetime (Feigin et al., 2018). Stroke is generally caused by genetic, environmental, socioeconomic, and vascular risk factors, and unhealthy habits such as low level of physical activity, poor nutrition, and alcohol or tobacco consumption (Avan et al., 2019; Boehme et al., 2017). To date, motor and language consequences of stroke have been most studied. Despite the significant impact of cognitive and emotional dysregulations on quality of life (QOL), social relationships, and return to work, these domains have not yet been given sufficient importance in post-stroke evaluation and rehabilitation processes (Slenders et al., 2020).

Depending on the nature of the stroke lesion, the cognitive and emotional post-stroke consequences can vary. Post-stroke depression affects up to 27% of stroke patients without a previous history of depression, and stroke patients who experience depression within the first 3 months have a 53% of probability of developing persistent depression (Liu et al., 2023). The presence of depressive symptoms following stroke hinders the rehabilitation process and has a strong negative impact on patients’ QOL (Ellis et al., 2010; Gaete & Bogousslavsky, 2008). Post-stroke depression has been linked to a more severe functional disability (Rabi Žikić et al., 2014), higher levels of anxiety (Schöttke & Giabbiconi, 2015), shorter interval to recurrence of ischemic stroke (Sibolt et al., 2013), and higher risk of mortality (Robinson & Jorge, 2016).

Frontostriatal strokes typically involve damage to the prefrontal cortex and/or subcortical structures of the basal ganglia, including the caudate nucleus, putamen, and globus pallidus, as well as the white matter tracts that interconnect these regions. Lesions affecting this circuit are particularly relevant for reward processing, decision-making, and goal-directed behavior (Averbeck & O’Doherty, 2022; Rolls et al., 2020). Damage may arise from either ischemic or hemorrhagic stroke, although ischemic strokes are more prevalent, particularly in subcortical territories supplied by penetrating arteries such as the lenticulostriate branches (Bogousslavsky et al., 1988). Laterality of the lesion may influence outcomes, particularly for cognitive and affective functions; however, evidence is mixed, and both left- and right-sided frontostriatal damage have been associated with deficits in reward sensitivity and depressive symptoms (Hama et al., 2011; Rochat et al., 2013). Disruptions to white matter integrity in frontostriatal circuits – especially in the anterior limb of the internal capsule and fronto-subcortical pathways – can result in widespread network dysfunction, even beyond the lesioned site (Guggisberg et al., 2019a, 2019b). Given the distributed nature of these networks, frontostriatal stroke is often associated with executive dysfunction, apathy, and impaired reinforcement learning (Wagner et al., 2023). Although frontal/anterior strokes and injuries in the basal ganglia have been linked to higher rates of post-stroke depression (Das & Rajanikant, 2018; Jaracz et al., 2014; Robinson & Jorge, 2016), depressive symptoms in stroke populations remain significantly under-evaluated and therefore under-treated (Medeiros et al., 2020).

Reward sensitivity is a dimension that has been proposed as a potential core factor in the development and persistence of mental disorders, especially depression (Proudfit, 2015). Reward sensitivity indicates the extent to which a person is driven by and actively pursues rewarding stimuli (Berridge & Robinson, 2003). This brain-behavior dimension is part of the Positive Valence Systems of the NIMH Research Domain Criteria (RDoC), which covers responses to positive motivational conditions, such as reward seeking, consummatory behavior, and reward/habit learning (National Institute of Mental Health (NIMH), 2018). The main goal of the NIMH RDoC is to uncover pathophysiological mechanisms that traverse, or are common to, various psychiatric disorders (Insel et al., 2010). As a transdiagnostic factor, reward sensitivity has been linked to various psychopathological profiles, including anhedonia. Anhedonia refers to a reduced capacity to experience pleasure and is a core symptom of major depressive disorder (MDD). Beyond hedonic blunting, recent research highlights a motivational subtype of anhedonia characterized by a diminished willingness to exert effort to obtain rewards (Treadway & Zald, 2013). This reduced responsiveness to reward – particularly in the anticipatory or effortful phases – has been associated with functional alterations in frontostriatal circuits in individuals with unipolar depression (Borsini et al., 2020). In contrast, elevated reward sensitivity has been linked to hypo/manic symptoms, marked by excessive approach motivation in bipolar disorder (Nusslock & Alloy, 2017). Research has consistently reported a positive association between reward sensitivity and depressive symptoms (Berry et al., 2019), as well as with MDD diagnosis (Foti et al., 2014), while lower reward sensitivity is often observed in individuals with high levels of anhedonia (Slaney et al., 2023).

Neuroimaging studies have provided insights into the neural underpinnings of reward-processing deficits in both MDD and stroke. In MDD patients, striatal hypoactivation coupled with dorsolateral prefrontal cortex (DLPFC) hyperactivation contributes to general anhedonia deficits (Borsini et al., 2020), with disrupted cortico-striatal further accentuating these impairments (Höflich et al., 2018; Keren et al., 2018; Zhang et al., 2013). Notably, decreased resting-state functional connectivity (rsFC) between the ventromedial prefrontal cortex (vmPFC), and left-lateralized frontoparietal regions, along with increased rsFC in right-lateralized regions, has been linked to anhedonia severity (Downar et al., 2014). A similar neural pattern is evident in stroke patients, where reduced reward sensitivity is associated with higher self-reported apathy, frontal hypoactivation, and reduced connectivity among frontal and temporoparietal regions (Rochat et al., 2013; Wagner et al., 2023). Intact dorsal striatum function, on the other hand, is linked to better responsiveness to incentives (Li et al., 2022).

Deficits in reward learning, a key component of anhedonia, are particularly linked to orbitofrontal cortex (OFC) hypoactivation (Borsini et al., 2020). Reward learning engages a broad neural network, including (Averbeck & O’Doherty, 2022): the OFC, which encodes reward value and modulates behavior through its projections to learning and emotion-regulation areas (Rolls et al., 2020); the DLPFC, which combines past decisions and rewards to update expected outcomes and optimize decision-making (Barraclough et al., 2004); the posterior parietal cortex (PPC), which integrates sensory, attentional, and reward-related signals to compute decision variables that guide actions (Sugrue et al., 2004); the premotor cortex, which encodes motor planning signals modulated by the subjective desirability of options (Pastor-Bernier & Cisek, 2011); and the striatum, which processes reward values and guides action selection by integrating dopaminergic signals that modulate cortico-striatal plasticity (Samejima et al., 2005). Together, these regions form a network that supports adaptive decision-making, playing a role in both MDD and post-stroke depression (Breitenstein et al., 1998; Shi et al., 2017). Notably, post-stroke emotional dysregulation is not solely attributable to focal lesions but also to white-matter disruptions affecting large-scale neural networks (Guggisberg et al., 2019a). Connectivity analyses highlight frontostriatal dysfunction in anhedonia, with depression patients failing to sustain reward-related activation patterns (Heller et al., 2009).

The study of cognitive and affective stroke consequences still struggles with the limitation lesion heterogeneity (Biesbroek & Biessels, 2023; Woranush et al., 2021). To the best of our knowledge, although previous studies have examined reward sensitivity and its neural correlates in stroke populations, several limitations remain unaddressed. Prior work has typically used heterogeneous stroke samples, limiting anatomical specificity (Hama et al., 2011; Rochat et al., 2013; Wagner et al., 2023). Wagner et al. (2023), for example, explored structural and functional alterations related to reward processing after stroke, but did not focus on reward learning per se, nor on frontostriatal circuits specifically. The present study builds on this literature by targeting a well-defined population of chronic frontostriatal stroke patients, a lesion profile directly implicated in reward-learning circuits. Furthermore, our study employs a reinforcement-learning task sensitive to reward-based decision-making deficits and explores resting-state functional connectivity in cortical areas relevant for reward valuation (OFC), cognitive control (DLPFC), and action preparation (PMC and SMA). In doing so, the current work addresses a critical gap in the understanding of post-stroke reward processing by linking focal frontostriatal damage to both behavioral impairments and network-level disruptions. Particularly, we focused on anhedonia-related reward-learning deficits, recording the rsFC of cortical areas involved in reward valuation and selection during reinforcement learning (Averbeck & O’Doherty, 2022) through a probabilistic reversal learning task. Based on previous findings, this study hypothesized that stroke patients would show lower reward sensitivity compared to healthy controls, and that these impairments would be associated with higher depressive symptoms and lower rsFC between the OFC, DLPFC, PPC, and premotor areas.

## Materials and methods

### Participants

For the aim of the study, N = 54 participants were recruited. Of these, *n* = 32 were patients who had suffered a frontostriatal stroke in the chronic phase, from the Neurology Unit of the Torrecardenas University Hospital, in Almeria, Spain and La Paz University Hospital, in Madrid, Spain. A neurologist introduced them to the study and ensured that they met the eligibility criteria. The inclusion criteria were: (1) a history of stroke between 6 months and 5 years prior, and (2) having undergone an MRI or computerized axial tomography (CAT), (3) revealing lesions in the prefrontal cortex (dorsolateral, ventrolateral, orbital and/or prefrontal gyrus) or in regions directly connected to these areas, including both cortical (precentral gyrus) and subcortical (basal ganglia) regions (Online Supplementary Material (OSM) 1 details the characteristics of each patient’s lesion), and (4) the absence of a diagnosed cognitive impairment. Additionally, we recruited *n* = 22 healthy participants who had no genetic, neurological, severe, or incapacitating mental disorders, or any history of substance abuse, assessed through clinical interview with a health psychologist. These participants were recruited from the community by word of mouth. All participants were adults between 18 and 55 years of age, an upper age limit chosen to minimize the potential confounding effects of age-related cognitive decline (Salthouse, 2004). There were no significant differences between the two groups with regard to gender, age, years of education, or annual income. All participants were volunteers and provided signed informed consent, and the information was treated under the Organic Law 3/2018 of 5 December, on the Protection of Personal Data and Guarantee of Digital Rights, which adapts the Spanish legal system to Regulation (EU) 2016/679 of the European Parliament and of the Council of 27 April 2016.

After ensuring the eligibility criteria, the neurologists in charge at the Neurology Unit assessed the degree of disability in daily activities with the Modified Rankin Scale (mRS) (Banks & Marotta, 2007), the stroke severity with the National Institutes of Health Stroke Scale (NIHSS) (Brott et al., 1989), and collected information about the stroke characteristics. Medication was registered although not altered.

Then, all participants underwent a clinical interview to collect demographic information and completed the following questionnaires: the International Physical Activity Questionnaire (IPAQ) (Mantilla-Toloza & Gómez-Conesa, 2007) to assess healthy habits and the 14-point Mediterranean Diet Adherence Screener (MEDAS) (García-Conesa et al., 2020). Finally, the self-perceived QOL was assessed with the Spanish version of the World Health Organization Quality of Life – BREF (WHOQOL-BREF) (Lucas-Carrasco, 2012). Table 1 summarizes the sociodemographic and clinical characteristics of both groups.

**Table 1:** Demographics and clinical measures of the sample

	Control	Stroke
	(*n* = 22)	(*n* = 32)
Demographics		
*n* (%) women	10 (45.5)	13 (40.6)
Age, *mean* ± *SD*	46.1 ± 7.5	46.2 ± 1.7
Annual income (€)^a^, *mean* ± *SD*	21.650 ± 10.289	22.029 ± 23.398
Years of education, *mean* ± *SD*	13.91 ± 5.7	12.0 ± 4.7
Medication, *n (%)*		
*Antidepressant*	1 (4.5)	12 (37.5)**
*Anxiolytic*	3 (14.3)	6 (18.8)
Stroke characteristics		
Ischemic, *n* (%)		20 (62.5)
Months after stroke, *mean* ± *SD*	-	22.1 ± 13.4
Lateralization of the lesion, *n* (%)		
*Left hemisphere*	-	14 (43.8)
*Right hemisphere*	-	13 (40.6)
*Both hemispheres*	-	5 (15.6)
Lesion volume (cm^3^), *mean* ± *SD*		18.5 ± 13.7
Modified Rankin Scale, *mean* ± *SD*	-	1.5 ± 0.8
NIHSS at inclusion,* mean* ± *SD*		1.1 ± 1.8
Health habits		
IPAQ (MET-min/week), *mean* ± SD	3094.5 ± 3963.7	2822.1 ± 3137.74
MEDAS^a^*, mean* ± *SD*	9.5 ± 2.0	8.8 ± 2.5
Self-perceived quality of life		
WHOQOL-BREF, *mean* ± *SD*		
Physical	71.6 ± 9.9	51.5 ± 19.3***
Psychological	67.6 ± 9.0	56.5 ± 16.4**
Social Relations	62.3 ± 1 6.6	51.1 ± 24.9
Environment	62.0 ± 10.7	51.1 ± 15.1**

Group-level differences were assessed via t-tests and χ2 tests

^a^ Missing information for one stroke patient

^***^p <.001, **p <.01, *p <.05

*IPAQ* International Physical Activity Questionnaire, *MEDAS* Mediterranean Diet Adherence Screener, *NIHSS* National Institutes of Health Stroke Scale, *SD *standard deviation, *WHOQOL-BREF* World Health Organization Quality of Life – BREF

**Fig. 1:**
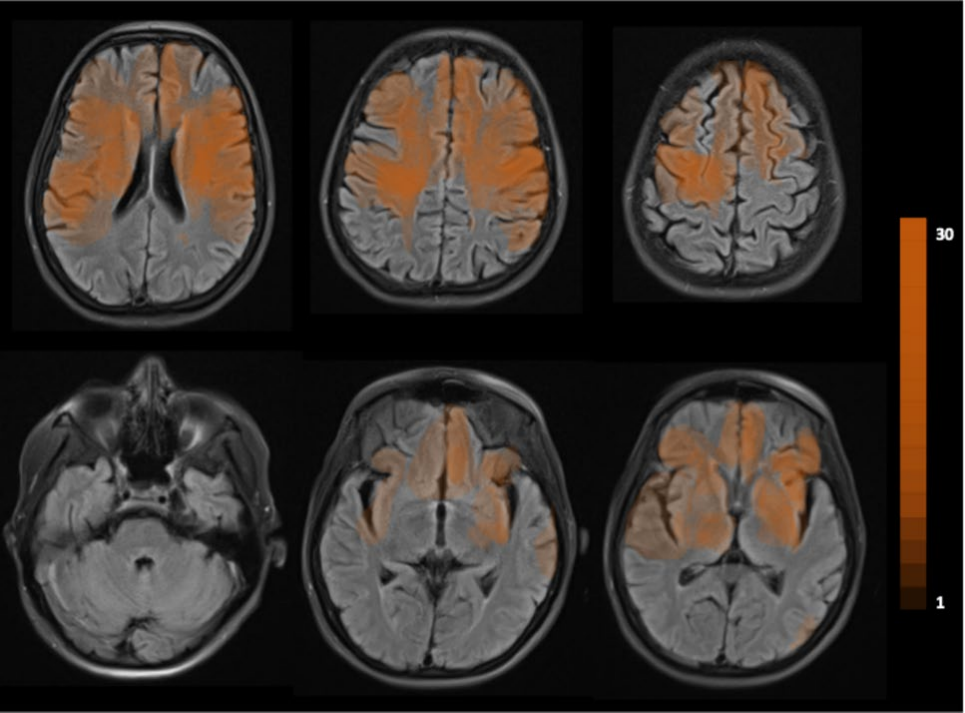
Distribution of lesions and the extent of overlap among patients. The color gradient reflected the number of patients with damage in each brain region (ranging from 1 to 30). Two patients (*n* = 2) had lesions that did not overlap with those of any other

A volumetric analysis of the brain lesions was conducted using the Vue PACS software (Carestream Health). Each lesion was semi-automatically segmented on axial cranial MRI slices under the supervision of two of the investigators to ensure anatomical accuracy. The segmented lesions were then semi-automatically co-registered, and their spatial overlap was computed. The degree of lesion overlap was visualized on a representative axial MRI template to illustrate the topographic distribution across cases (see Fig. 1).

### Materials

#### Adult Self-Report scale (ASR)

Depression was measured with a computerized Spanish version of the Adult Self-Report scale (ASR) (Achenbach et al., 2003). The ASR is a 126-item self-report questionnaire for adults (ages 18–59 year) that assesses adaptive functioning and problems, which is part of the Achenbach System of Empirically Based Assessment (ASEBA) taxonomy. For this study, we used the T-score of depressive problems of the DSM-oriented scales. The ASR was selected for its ability to assess a broad range of psychopathological symptoms through both dimensional and categorical measures (Rescorla & Achenbach, 2004), aligning with the aim to conduct an exploratory study of potential mental health problems resulting from stroke and to rule out the potential fulfilment of diagnostic criteria in the control group.

#### Probabilistic Reversal Learning Task (PRLT)

This task enables the evaluation of participants’ reward sensitivity (Bari et al., 2010; Isıklı et al., 2023) and cognitive flexibility towards the reversal of contingencies (Izquierdo et al., 2017; Jara-Rizzo et al., 2020). The task consisted of four blocks of 40 trials each, in which participants were instructed to earn as many points as possible by choosing between two different stimuli. The stimuli consisted of two squares, each of a different color, randomly alternating their positions. One of the squares was considered correct and would give 5 points, while the other was considered incorrect and would deduct five points. Additionally, participants would receive auditory and visual feedback associated with the reward or punishment. In the first two blocks, the correct color was in an 80/20% proportion, and in the last two blocks, it was in a 70/30% proportion. Before starting the experimental phase, participants performed a ten-trial test with an 80/20% proportion. After every 40 trials, the contingencies were reversed, and the stimulus with the highest probability of being correct became the one with the highest probability of being incorrect, and vice versa. The variables measured in this task were: (1) Accuracy, measured through the proportion of choices of the mostly correct square (hits), (2) reward sensitivity, measured through the proportion of trials in which participants chose the same stimulus after receiving positive feedback throughout the task and in each block (win-stay probability), and (3) punishment sensitivity, measured through the proportion of trials in which participants switched stimuli after receiving negative feedback throughout the task and in each block (lose-shift probability). Hence, these variables assess reward sensitivity and cognitive flexibility in a dynamic decision-making setting that includes gains and losses.

#### rsFC

Functional brain connectivity at rest was assessed using the fNIRS optical neuroimaging technique. A 16 × 16 setup was employed by simultaneously connecting two portable NIRSport 8 × 8 models (NIRx Medical Technologies, Berlin). This instrument enables real-time recording of hemodynamic changes in the cortical brain through sources that emit light and detectors that receive it. It emits two different wavelengths, 760 nm and 850 nm, allowing for the recording of both changes in the concentration of oxygenated hemoglobin (HbO) and deoxygenated hemoglobin (Hb). However, in this study, only the level of HbO was selected because it showed greater sensitivity to changes in cerebral blood flow (Duan et al., 2012). The registration pre-processing was carried out using the NIRS Brain AnalyzIR Toolbox (Santosa et al., 2018), starting with cleaning the channel saturation and missing values, and subsequently converting the data to optical density to obtain HbO values in the end.

**Fig. 2:**
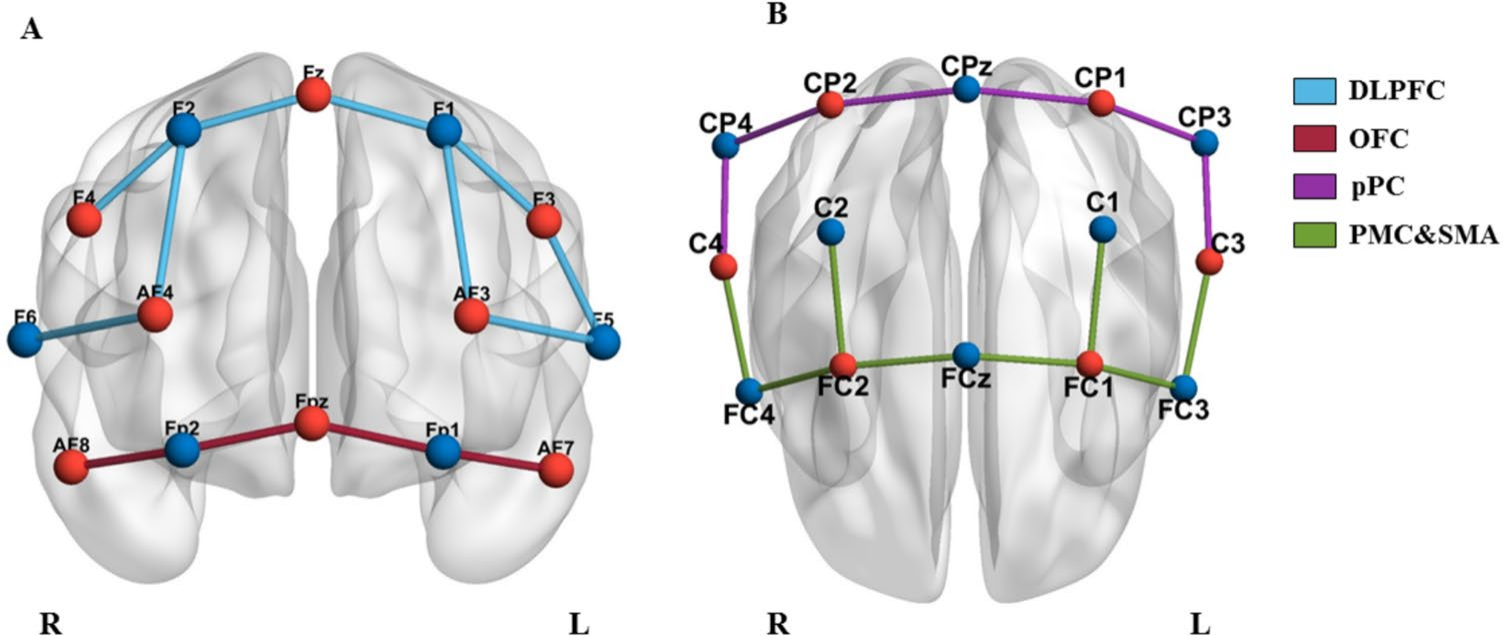
**A and B** The optodes and channels of the prefrontal cortex and the motor cortex, respectively. In both figures, the red color represents the 14 sources (F4, Fz, F3, AF4, AF3, AF8, Fpz, AF7, CP2, CP1, C4, C3, FC2 and FC1), the blue color represents the 14 detectors (F2, F1, F6, F5, Fp2, Fp1, CP4, CPz, CP2, C2, C1, FC4, FCz and FC3) and the lines represent (**A**) each of the 13 prefrontal cortex (blue and red) and (**B**) each of the 14 motor cortex channels (purple and green). The arrangement of the channels is algo displayed in Table 2. The optode placement corresponds to the international 10–10 international system for EEG. DLPFC = Dorsolateral prefrontal cortex, L = left hemisphere, OFC = orbitofrontal cortex, PMC&SMA = premotor cortex and supplementary motor area, pPC = posterior parietal cortex, R = Right hemisphere

The setup used allowed for the recording of hemodynamic activity from 27 channels, 13 of which were located in the prefrontal cortex and 14 in the motor cortex. The arrangement of the sources and detectors (both referred to as optodes) is shown in Figs. 2A and B.

To analyze functional connectivity, cross-correlations were performed between regions of interest (ROIs). These regions included the left and right orbitofrontal cortex (lOFC, rOFC), the left and right dorsolateral prefrontal cortex (lDLPFC, rDLPFC), the left and right posterior parietal cortex (lpPC, rpPC), and the left and right premotor cortex and supplementary motor area (lPMC&lSMA, rPMC&rSMA). To determine the connectivity value of each ROI, the average of each channel comprising it was calculated. The channels of each ROI and their location were obtained using the “fNIRS Optodes'Location Decider tool,” and are shown in Table 2. The mean distance between channel optodes was 35.6 mm (SD = 5.98).

**Table 2:** Regions of interest (ROIs) and coordinates of the fNIRS setup

ROI	Source	Detector	Distance	X	Y	Z
lOFC						
	AF7	Fp1	30	−33	59	−2
	Fpz	Fp1	30	−12	67	0
rOFC						
	Fpz	Fp2	31	13	67	0
	AF8	Fp2	30	34	59	−2
lDLPFC						
	F3	F1	29	−31	39	41
	F3	F5	29	−46	39	26
	AF3	F5	44	−39	50	17
	AF3	F1	44	−23	52	32
	Fz	F1	29	−9	41	50
rDLPFC						
	Fz	F2	29	10	41	50
	AF4	F2	44	22	52	33
	AF4	F6	45	40	50	16
	F4	F2	30	30	40	41
lpPC						
	CP1	CPz	38	−16	−50	72
	CP1	CP3	38	−39	−48	60
	C3	CP3	37	−52	−34	52
rpPC						
	C4	CP4	36	53	−35	52
	CP2	CP4	38	39	−49	60
	CP2	CPz	39	17	−50	73
lPMC&lSMA						
	FC1	FC3	36	−38	12	55
	FC1	FCz	36	−13	12	67
	FC1	C1	39	−26	−5	68
	FC3	C3	37	−50	−3	50
rPMC&rSMA						
	FC2	FC4	36	39	12	54
	FC2	FCz	36	14	13	66
	FC2	C2	40	27	−4	68
	C4	FC4	38	52	−4	48

Unit of measurement is mm

*lDLPFC* left dorsolateral prefrontal cortex, *lOFC* left orbitofrontal cortex, *lPMC&lSMA* left premotor cortex and left supplementary motor area, *lpPC* left posterior parietal cortex, *rDLPFC* right dorsolateral prefrontal cortex, *rOFC* right orbitofrontal cortex, *rPMC&rSMA* right premotor cortex and right supplementary motor area, *rpPC* right posterior parietal cortex

### Procedure

Participants provided informed consent and their sociodemographic and clinical data were collected through the interview and questionnaires. The assessment of rsFC with fNIRS was carried out for 9 min while participants were instructed to sit still, try to relax as much as possible, keep their eyes open, and look at a blank wall. After removing the fNIRS device, participants completed the PRLT task for approximately 10 min. Instructions were provided within the task and participants were asked if they had any questions before starting.

All procedures contributing to this work comply with the ethical standards of the relevant national and institutional committees on human experimentation and with the Helsinki Declaration of 1975, as revised in 2008. This study was approved by the bioethics committees of the Torrecardenas University Hospital and the human research committee of the University of Almeria.

### Statistical analysis

Statistical analyses were conducted using JASP (v. 0.17.3) (Amsterdam, Netherlands) and R software (R Core Team, 2022). t-tests were applied to assess group-level differences in depressive problems. When assumptions were violated, we applied robust statistics on 10% trimmed means and 2,000 bootstrap samples for better control of Type I error tests (bootstrap version of Yuen’s tests) (Field & Wilcox, 2017; Mair & Wilcox, 2020). We performed robust two-way mixed ANOVAs (Villacorta, 2019) to analyze the effect of Group (between-subjects factor: stroke vs. control) and Block (within-subjects factor: block 1 vs. block 2 vs. block 3 vs. block 4) on the number of hits, and the win-stay and lose-shift probabilities in the PRLT. For pairwise comparisons between blocks in the number of hits, t-tests were used when the assumption of normality was met; otherwise, robust alternatives were applied. All post hoc tests used the Benjamini–Hochberg correction for multiple comparisons.

To analyze the rsFC data of the ROIs, as several comparisons were made, Bayesian data analysis was performed to ensure better Type I error control (Kruschke, 2014). Evidence for H₁ was considered when BF₁₀ > 1, while evidence for H₀ was indicated by BF₁₀ < 1. BF₁₀ interpretation followed the categories proposed by Lee and Wagenmakers (2013): 1 < BF₁₀ < 3, anecdotal evidence for H₁; 3 < BF₁₀ < 10, moderate evidence; 10 < BF₁₀ < 30, strong evidence; 30 < BF₁₀ < 100, very strong evidence; and BF₁₀ ≥ 100, extreme evidence.

In variables where stroke patients differed from healthy participants, linear regression analysis was performed using win-stay probability (reward sensitivity) as the dependent variable. Additionally, to explore individual differences within the stroke group, we examined whether lesion size was associated with behavioral performance and depressive symptom scores. Given that lesion size did not follow a normal distribution (as tested by the Shapiro–Wilk test), we used Spearman’s rank-order correlations to assess its relationship with the number of hits, win-stay and lose-stay probabilities, and depressive problems. Pearson correlation coefficients were computed between the resting-state frontostriatal connectivity measure and the QOL subscale scores. The significance level was set at *p* ≤ 0.05.

## Results

**Fig. 3:**
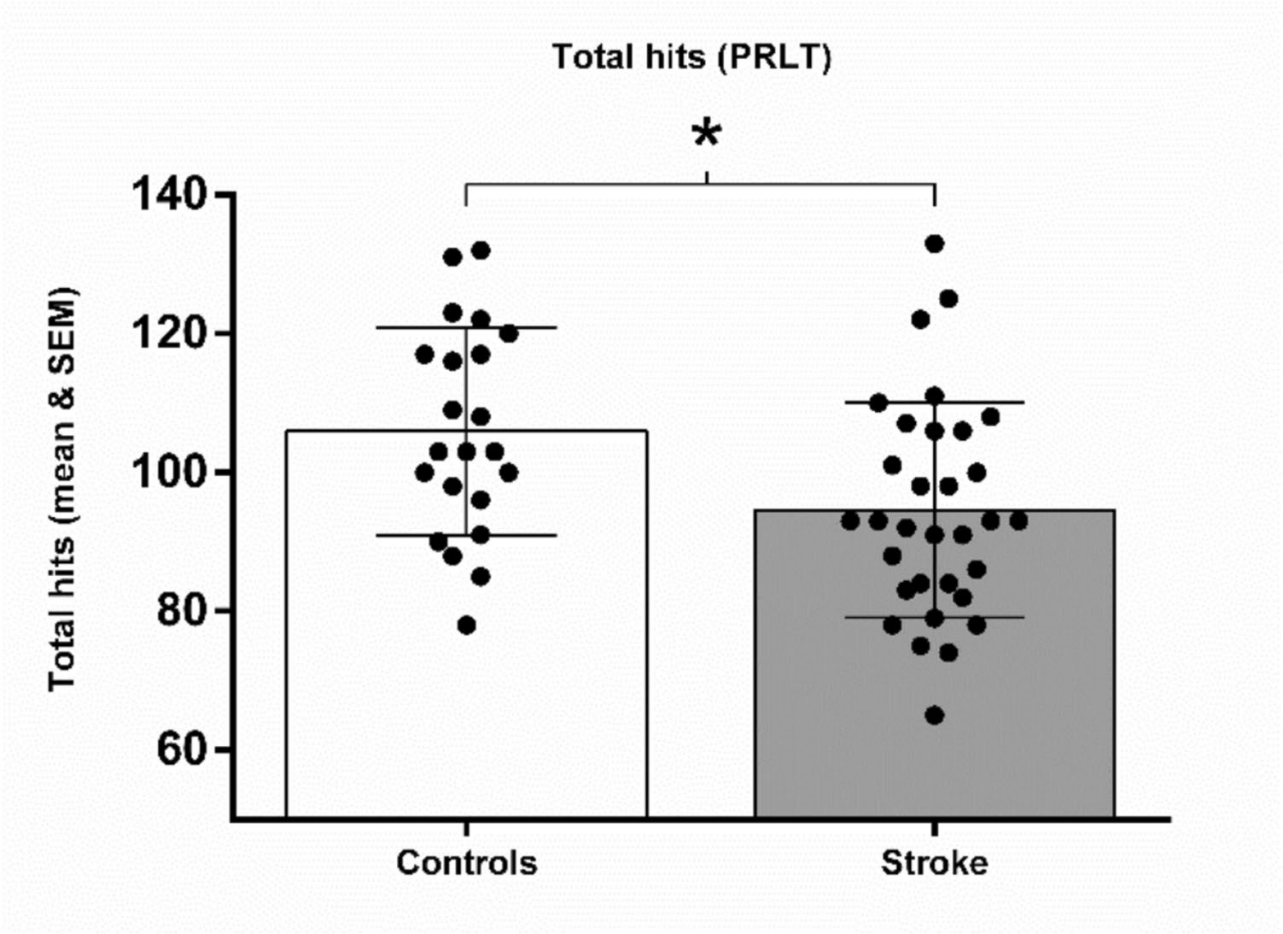
Mean and SEM of the total hits obtained by each group in the Probabilistic Reversal Learning Task (PRLT). * *p* ≤.050

A robust two-way mixed ANOVA on PRLT hits (Fig. 3) revealed significant main effects of group (T_WJ(1, 35.16)_ = 6.080; *p* = 0.020) and block (T_WJ(3, 30.93)_ = 11.945; *p* < 0.001), but no interaction effect was found (T_WJ(3, 30.93)_ = 2.647; *p* = 0.082). The analysis of simple effects revealed that stroke patients obtained significantly lower hits than controls on the complete task (T_WJ(1, 35.16)_ = 6.080; *p* = 0.019, δ^1^ = −1.852). Block 1 yielded the highest number of hits (M = 28.88), suggesting greater accuracy in the initial 80/20 acquisition phase. A paired-sample t-test comparing average hits in the 80/20 blocks (blocks 1 and 2) versus the 70/30 blocks (blocks 3 and 4) revealed a significant effect of uncertainty, with participants performing better under lower uncertainty: t(31) = 3.01, *p* = 0.005. Specifically, participants achieved significantly more hits in block 1 than in block 3 (t(31) = 4.83, *p* < 0.001), whereas no significant difference was found between block 2 and block 4 (t(31) = 0.35, *p* = 0.729). These findings support the expected effect of uncertainty and reversal on task performance, thus contributing to task validation.

**Fig. 4:**
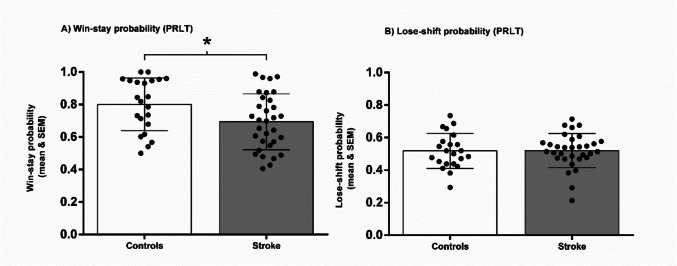
A. Mean and SEM of the win-stay probability by each group in the Probabilistic Reversal Learning Task (PRLT). **B.** Mean and SEM of the lose-shift probability by each group in the PRLT. * *p* ≤.050

Regarding the win-stay probability obtained in the PRLT (Fig. 4A), a robust two-way mixed ANOVA revealed a significant main effect of group (T_WJ(1, 36.90)_ = 5.624; *p* = 0.018), but not block (T_WJ(3, 34.45)_ = 2.606; *p* = 0.087) nor interaction effects (T_WJ(3, 34.45)_ = 2.005; *p* = 0.114). The analysis of simple effects revealed that stroke patients exhibited significantly lower win-stay probability than controls on the complete task (TWJ(1, 36.9) = 5.624; p = 0.023, δ = 2.245). In lose-shift probability (Fig. 3B), analyses showed no significant group (T_WJ(1, 30.05)_ = 0.0236; *p* = 0.620) nor block (T_WJ(3, 27.18)_ = 0.440; *p* = 0.727) effects, or interaction effect (T_WJ(3, 27.18)_ = 0.289; *p* = 0.848).

**Fig. 5:**
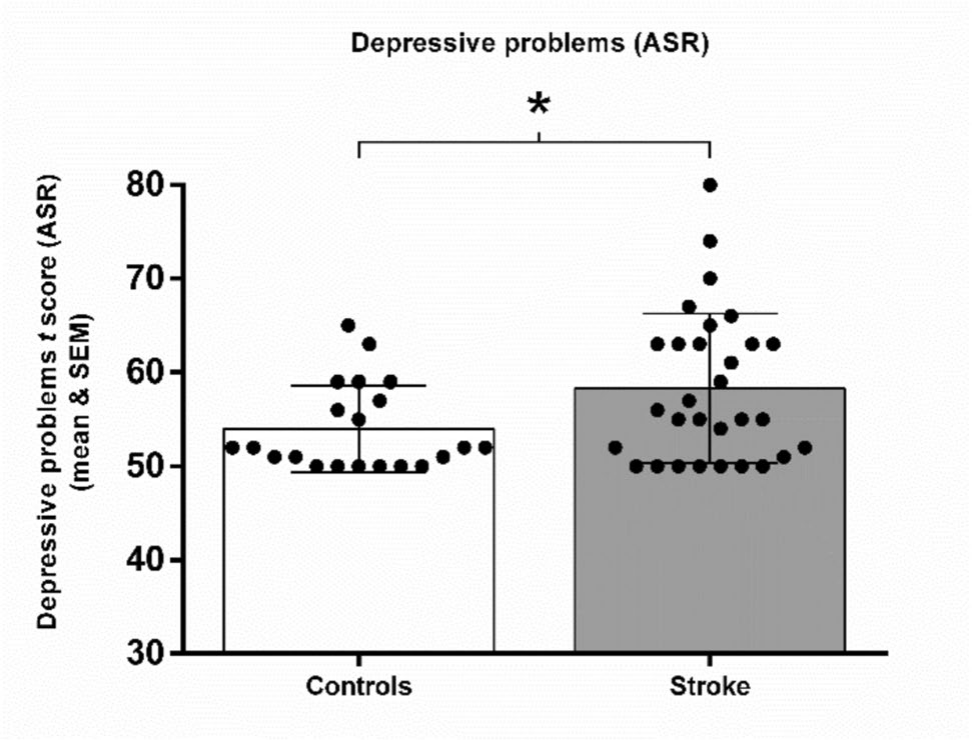
Mean and SEM of the *t* score of depressive problems obtained by each group in the Adult Self-Report scale (ASR). * *p* ≤ 050

Analyses of depressive problems were performed for 30 participants with stroke and 21 controls. Data were missing for three participants. Robust t-tests (bootstrap version of Yuen’s tests) revealed that stroke patients had significantly higher depressive problems compared to healthy controls (M_diff_ = −3.9975 [−7.549, −0.445], Yt = −2.250, *p* = 0.025) (Fig. 5).

In stroke patients, results indicated no significant correlations between lesion size (cm^3^) and any of the previous variables: hits, ρ(25) = 0.33, *p* = 0.066; win-stay probability, ρ(25) = 0.12, *p* = 0.502; lose-stay probability, ρ(25) = –0.03, *p* = 0.878; or depressive problems, ρ(25) = –0.19, *p* = 0.316.

**Fig. 6:**
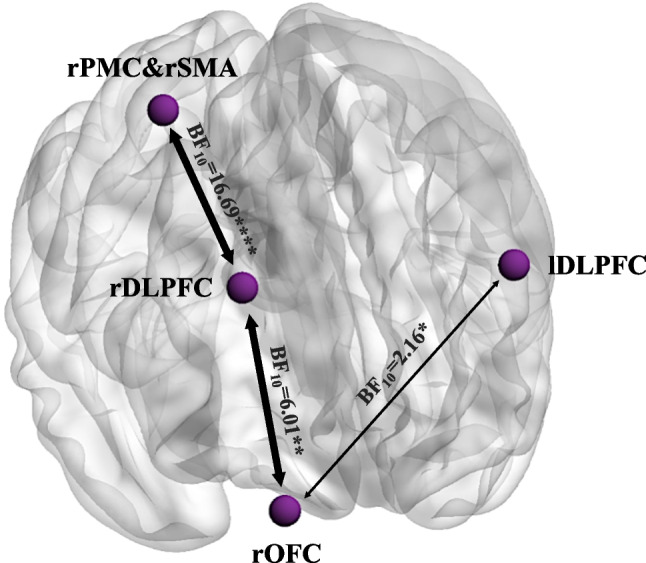
H1 evidence in support of differences between the stroke patients and healthy controls in the rsFC. * = anecdotal, ** = moderate, *** = strong, **** = very strong, ***** = extreme evidence for H1

rsFC analyses were performed over 31 stroke patients and 22 controls. In the group comparisons of resting-state functional connectivity (rsFC), a Bayesian ANCOVA controlling for depressive problems revealed strong evidence in favor of the alternative hypothesis for reduced connectivity in stroke patients between the rDLPFC and the rPMC&rSMA, BF₁₀ = 16.69: stroke patients (M = 0.337, 95% CI [0.208, 0.466]) showed lower connectivity compared to controls (M = 0.583, 95% CI [0.457, 0.710]). Moderate evidence was also observed for the rOFC-rDLPFC connection, BF₁₀ = 6.01, with stroke patients (M = 0.225, 95% CI [0.112, 0.339]) again showing lower connectivity than controls (M = 0.419, 95% CI [0.301, 0.537]). Anecdotal evidence for a group difference was also observed in the connectivity between the rOFC and lDLPFC with BF₁₀ = 2.16, showing stroke patients showed lower connectivity (M = 0.126, 95% CI [0.067, 0.185]) compared to controls (M = 0.236, 95% CI [0.148, 0.324]) (Fig. 6). All other comparisons provided no evidence for group differences. The covariate depressive problems was included in all models but did not significantly predict rsFC in any of the comparisons (*p* > 0.05).

**Fig. 7:**
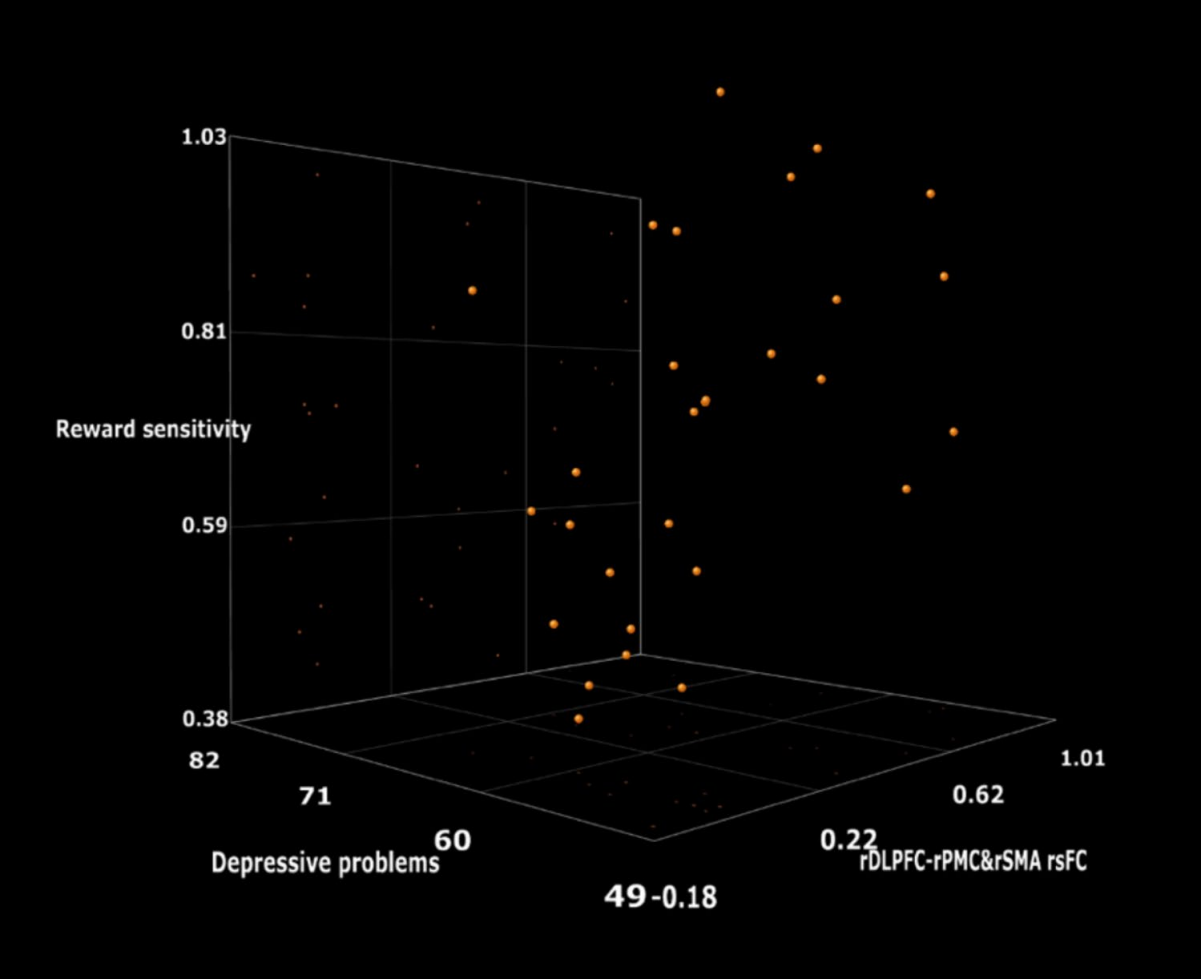
Three-dimensional scatter plot (MiaBella, LLC, Denver) of the reward sensitivity prediction model that shows depressive problems and the rDLPFC-rPMC&rSMA rsFC as significant predictors of the reward sensitivity. Visualization of the interactive scatter plot is available at https://miabellaai.net/index.html by introducing the data set “3 dscatterplot” included in https://osf.io/bs329/?view_only=ffabefcecb5549ac8dccb77b7e040c17

A multiple regression was run in stroke patients to predict reward sensitivity including as predictors depressive problems and the rOFC-rDLPFC, rOFC-lDLPFC and rDLPFC-rPMC&rSMA rsFC using standardized measures. Data analysis was performed over 29 stroke patients (one had missing data in depressive problems and neuroimaging, and two had missing data in neuroimaging). Correlation analyses revealed no significant associations between frontostriatal connectivity and any of the QOL subscales scores. These results suggest that the observed connectivity alterations are not directly related to self-perceived QOL or functional disability in this sample. To control for potential confounding effects, the following covariates were included in the model: sex, age, years of education, time since stroke (in months), lesion size (in cm^3^), and functional impairment as measured by the Modified Rankin Scale. None of the covariates showed statistically significant associations with reward sensitivity (*p*s > 0.16). The results showed a significant model R_2_ = 0.256, F(4, 45) = 3.671, *p* = 0.011. The individual predictors were examined further and indicated that depressive problems (β = −0.360, *p* = 0.001) and the rDLPFC-rPMC&SMA rsFC (β = 0.530, *p* = 0.011) were significant predictors but rOFC-rDLPFC and rOFC-lDLPFC were not (β = −0.340, *p* = 0.130 and β = −0.181, *p* = 0.304, respectively) (Fig. 7).

## Discussion

The present study found that compared to healthy controls, patients with chronic frontostriatal stroke displayed lower reward sensitivity, higher levels of depressive problems, and lower rOFC-rDLPFC, rOFC-lDLPFC and rDLPFC-rPMC&rSMA rsFC. Furthermore, results showed that in stroke patients, a low reward sensitivity was predicted by higher depressive problems and lower rDLPFC-rPMC&rSMA rsFC.

Results revealed that frontostriatal stroke patients in comparison with healthy controls displayed lower hits in general across the PRLT, with no block interaction, presenting lower reward sensitivity by means of a lower win-stay probability. This is in accordance with previous literature describing a lower reward sensitivity in chronic post-stroke patients (Li et al., 2020; Quattrocchi & Bestmann, 2014; Rochat et al., 2013). As a transdiagnostic dimension, reward sensitivity has been associated with depressive symptoms (Berry et al., 2019; Pulcu et al., 2023) but also alcohol consumption (Booth & Hasking, 2009), excessive eating and compulsive buying (Davenport et al., 2012), psychotic disorders (Le et al., 2022), and drug addiction (Volkow et al., 2010). Hence, the presence of a low reward sensitivity might increase the risk of developing comorbid pathologies and negatively affect the emotional state of the stroke patient (Ashaie et al., 2023), which hinders the rehabilitation progress. The present results of the regression model are in accordance with Potsch and Rief (2023), who found that reduced reward sensitivity seems to be a consequence of psychopathological symptoms, especially depression. Hence, evaluations in reward sensitivity and, when necessary, rehabilitation procedures on post-stroke patients, especially with frontostriatal lesions, should cover this dimension. In this sense, mental imagery training has been shown to increase reward sensitivity while reducing depressive symptoms (Linke & Wessa, 2017), and therapy sessions of positive affect treatment (PAT) have been shown to improve reward sensitivity in adults with moderate-to-severe depression (Craske et al., 2023).

Higher levels of depression in stroke and its high disruption into social, work, and daily life activities have been largely replicated in the literature (Medeiros et al., 2020). This work further supports the relevance of depression in post-stroke patients, especially when the frontostriatal pathway is damaged. Henceforth, these results corroborate the necessity of including depression in early evaluation and follow-ups of stroke evolution, and to address its intervention among rehabilitation treatments. In fact, it is known that the reduction of post-stroke depression during the initial months following a stroke is linked to a more significant improvement in activities of daily living functionality compared to persistent depression (Chemerinski, Robinson, & Kosier, 2001). Among the treatments for depressive problems in stroke, research has demonstrated the efficacy of early intervention using tricyclic antidepressants and selective serotonin receptor inhibitors (Richter et al., 2021). In our sample, 37.5% of the patients were taking antidepressants at the time of evaluation. Nevertheless, these pharmaceutical treatments have certain disadvantages, which have led to the emergence of alternative strategies like non-invasive brain stimulation, psychotherapy, and rehabilitation therapy, which are increasingly recognized for their positive outcomes in addressing post-stroke depression (Hao et al., 2023; Sarkar et al., 2021).

The lower performance in stroke patients in the PRLT was evidenced by a low reward sensitivity rather than an altered punishment sensitivity. To shift from a non-rewarding or punishing stimulus guides behavior, as it is caused by a universal avoidance that occurs regardless of the penalty magnitude (Kubanek et al., 2015). However, no differences were found in the lose-shift probability between patients and controls. These results are congruent with previous literature in frontal stroke patients, where specifically patients with OFC lesions showed no differences in the lose-shift frequency during the PRLT but presented higher win-shift behavior compared to healthy controls (Tsuchida et al., 2010).

We found lower rOFC-rDLPFC and rOFC-lDLPFC rsFC in stroke patients in comparison with controls. These results are congruent with the existing literature, as the connectivity of the OFC and the DLPFC has been shown to be altered in reward-related processing in major depressive disorder (Zhang et al., 2013) and especially in stroke patients (Siegel et al., 2016). The OFC plays a role in assigning emotional and motivational significance to stimuli, while the DLPFC contributes to cognitive control processes in response to salient stimuli. Both regions interact within the salience network, which is involved in detecting and integrating relevant internal and external stimuli to guide behavior (Menon, 2015; Seeley et al., 2007). An fMRI study found that stroke patients with low positive reward sensitivity also exhibited lower activation in the putamen, pallidum, thalamus, frontal and prefrontal cortices, and cerebellum when compared with controls (Lam et al., 2016), and Tsuchida et al. (2010) found that performance in the reversal task was particularly related to lesions in the OFC. The connectivity between the rOFC and the DLPFC has been associated with various behaviors, including decision-making (Hampshire et al., 2010) and emotion regulation (Ochsner et al., 2004). Moreover, a neuromodulation study has shown that the interaction of the rOFC and the lDLPFC is related to decision-making processes involving risk (Nejati et al., 2018). All these findings highlight reductions in connectivity between the OFC and DLPFC as a potential factor contributing to post-stroke deficits.

We found strong evidence in support of a lower rDLPFC-rPMC&rSMA rsFC of stroke patients in comparison to controls. Moreover, based on the standardized beta coefficients of the regression model, the rDLPFC-rPMC&rSMA rsFC emerged as a stronger predictor of reward sensitivity than depressive symptoms in stroke patients. Hence, this connectivity might be a key element for the lower motivation for the positive reward, as the DLPFC is part of the attentional network that is connected to the anterior cingulate cortex (ACC) involved in motivation, which together projects to the SMA and motor cortex for the sequencing motivated action (Posner & Petersen, 1990). Moreover, the PMC has shown an abnormal activity in major depressive disorder (Liu et al., 2021) and an involvement in the reward processing mechanism in depression (Rizvi et al., 2013). Hence, these results highlight that assessing networks and tailoring rehabilitation accordingly holds great promise for optimizing the effectiveness of specific interventions and improving overall outcomes following a stroke (Guggisberg et al., 2019b). Although our sample included only stroke survivors with relatively mild functional impairment, participants nonetheless reported lower perceived QOL across multiple dimensions. We examined whether QOL outcome could account for the observed frontostriatal connectivity alterations. However, our analyses did not reveal any significant associations, suggesting that these neural changes are not directly driven by self-reported well-being. Importantly, the group differences in frontostriatal functional connectivity remained significant after controlling for depressive symptoms, which were higher in the stroke group. This suggests that the observed connectivity alterations reflect lesion-related neural disruption, rather than being secondary to mood symptoms.

Although we initially hypothesized reduced connectivity involving PPC, our results did not reveal significant alterations in this region. One possibility is that the PPC’s contribution to reward learning is more task-dependent, and less apparent in resting-state functional connectivity (Levy & Glimcher, 2012). Moreover, parietal regions may show greater resilience to focal disruption due to compensatory mechanisms and distributed network properties (Corbetta et al., 2005). Finally, the alterations observed in stroke patients may predominantly involve prefrontal regions – such as the OFC and DLPFC and their connection to motor regions – which play a central role in reward valuation and goal-directed behavior (Averbeck & O'Doherty, 2022).

The present study has certain limitations. Although the regression model revealed that depressive symptoms (β = −0.360) and rsFC between the rDLPFC and rPMC&rSMA (β = 0.530) significantly predicted win-stay probability, these coefficients also suggest that additional factors may influence reward sensitivity in stroke patients. Future research should explore other potential contributors to fully understand the mechanisms underlying this effect. Moreover, the proportion of variance explained by the model was modest (R_2_ = 0.256), highlighting the need to consider additional predictors in future research. fNIRS is limited to measuring hemodynamic responses in cortical surface regions and does not allow for the assessment of subcortical structures. As a result, we were unable to capture activity in regions such as the striatum, which is also part of the reward system or limbic structures related to anhedonia and depression (Samejima et al., 2005). Also, future studies employing multimodal imaging with higher spatial resolution and larger sample sizes may help to further disentangle the specific contribution of lesioned versus non-lesioned tissue in functional connectivity patterns after stroke. Some of the participants were under stable pharmacological treatment, no washout procedure was applied, and medication regimens varied in type and dosage due to medical reasons. Nevertheless, the stroke population still showed higher depressive symptoms compared to controls. Given the relatively small sample size and the heterogeneity in medication type, dosage, and posology, conducting a sensitivity analysis would have lacked statistical power and may have introduced further noise and misinterpretation; however, this heterogeneity may have influenced reward-related connectivity patterns and represents a limitation that future studies with larger samples should address more systematically. Further research could also explore whether alterations in reward sensitivity and connectivity relate to functioning and QOL, particularly in stroke populations with a broader range of impairment severity and including older adults, where age-related cognitive decline may interact with post-stroke symptoms. This procedure could be also replicated in a sample of patients with post-stroke depression, including a non-lesioned control group with clinical depression, to further explore the specific effects of stroke and depression on reward sensitivity.

The present findings provide valuable insight into the neurobiological mechanisms that may underlie post-stroke depression, revealing how disruptions in reward circuitry could impact decision-making and motivation. The observed effects on reward sensitivity suggest that stroke-induced changes in brain connectivity, particularly within the prefrontal cortex, may exacerbate emotional and cognitive deficits, making rehabilitation efforts more challenging. From a rehabilitation perspective, understanding how stroke lesions affect reward processing can inform more targeted therapeutic interventions. For example, interventions that focus on improving motivation and decision-making could be particularly beneficial for stroke patients with significant reward sensitivity impairments. Furthermore, addressing depressive symptoms alongside cognitive and motor rehabilitation could help optimize recovery outcomes.

In conclusion, stroke patients exhibited lower reward sensitivity, greater depressive problems, and reduced rsFC compared to healthy controls. Specifically, decreased rsFC was found between the rOFC and the lDLPFC, the rOFC and the rDLPFC, and between the rDLPFC and the rPMC&rSMA. Moreover, reward insensitivity was significantly predicted by increased depressive symptoms and reduced rsFC in rDLPFC-rPMC&rSMA, highlighting the combined influence of affective symptoms and disrupted prefrontal-premotor connectivity on reinforcement learning impairments. This work underscores the relevance of addressing reward sensitivity in the assessment of post-stroke patients, supports the necessity of increasing the attention towards depression along the post-stroke evolution, and underlines rsFC as a valuable tool for characterizing abnormalities in connectivity in stroke patients.

## Supplementary Information

Below is the link to the electronic supplementary material.

Supplementary file1 (DOCX 18 KB)

## Data Availability

The data that support this study are openly available at https://osf.io/bs329/?view_only=ffabefcecb5549ac8dccb77b7e040c17.

## Footnotes

1 The explanatory measure of effect size (ξ), a robust generalization of Pearson’s correlation allowing assumptions deviations and unequal sample sizes. Analogue to Cohen’s *d*, ξ =.15,.35 and.50 roughly correspond to small, medium, and large effect sizes (Field & Wilcox, 2017; Rand & Tian, 2011).
