# Supplementary material for: Lower reward sensitivity in frontostriatal stroke: Influence of depression and resting-state functional connectivity
Source: Cogn Affect Behav Neurosci. 2025 Jun 6;25(6):1779–94. doi: 10.3758/s13415-025-01318-9 (PMC12615514; doi:10.3758/s13415-025-01318-9)
Supplement: Supplementary file 1 — Supplementary file1 (DOCX 18 KB) [file 13415_2025_1318_MOESM1_ESM.docx]

# **Supplementary material**

**Supplementary material 1.** Characteristics of each patient’s lesion. Main characteristics of the brain lesions.

| Patient | Stroke type | Side | Volume  (cm^3^) | Lesion topography | | | | | |
| --- | --- | --- | --- | --- | --- | --- | --- | --- | --- |
|  |  |  |  | DL | VM | OF | ACC | Caudate | Lenticular |
| 1 | Ischemic | Left | 24.3 |  | X |  | X |  |  |
| 2 | Ischemic | Left | 7.2 | X |  | X |  | X | X |
| 3 | Ischemic | Left | 8.3 | X |  |  |  |  |  |
| 4 | Ischemic | Left | 24.7 | X |  |  |  |  |  |
| 5 | Ischemic | Right | 36.1 |  |  | X |  | X | X |
| 6 | Ischemic | Right | 4.3 | X |  |  |  |  |  |
| 7 | Ischemic | Left | 6.9 | X |  |  |  |  |  |
| 8 | Ischemic | Bilateral | 52.5 | X | X |  | X | X |  |
| 9 | Hemorrhagic | Left | 50.3 | X |  |  |  | X | X |
| 10 | Ischemic | Left | 13.6 |  | X |  |  |  |  |
| 11 | Hemorrhagic | Left | 27.6 | X |  |  |  |  |  |
| 12 | Hemorrhagic | Right | 9.6 | X |  |  |  |  |  |
| 13 | Ischemic | Left | 3.5 |  | X |  | X | X |  |
| 14 | Ischemic | Right | 7.6 | X |  |  |  |  |  |
| 15 | Ischemic | Left | 15.3 | X |  |  |  |  |  |
| 16 | Hemorrhagic | Right | 13.9 |  |  |  |  |  |  |
| 17 | Hemorrhagic | Right | 7.9 |  |  |  |  |  | X |
| 18 | Ischemic | Left | 7.3 |  | X |  |  |  |  |
| 19 | Ischemic | Right | 6.7 | X |  |  |  |  |  |
| 20 | Ischemic | Right | 49.2 | X |  |  |  |  | X |
| 21 | Ischemic | Left | 19.7 | X |  |  |  | X | X |
| 22 | Ischemic | Left | 16.4 | X |  | X | X |  |  |
| 23 | Hemorrhagic | Bilateral | 10.5 | X |  | X | X |  |  |
| 24 | Hemorrhagic | Left | 35.2 | X |  |  |  |  |  |
| 25 | Hemorrhagic | Right | 25.1 | X |  |  |  |  |  |
| 26 | Hemorrhagic | Right | 11.7 | X |  |  |  |  |  |
| 27 | Hemorrhagic | Left | 28.6 | X |  |  |  |  |  |
| 28 | Ischemic | Bilateral | 20.2 | X |  |  | X |  |  |
| 29 | Hemorrhagic | Right | 3.2 | X |  |  |  |  |  |
| 30 | Hemorrhagic | Right | 13.6 | X |  |  |  |  |  |
| 31 | Ischemic | Right | 11.7 | X |  |  |  |  |  |
| 32 | Ischemic | Left | 20.3 | X | X |  |  |  |  |

*Note.* ACC: Anterior cingulate cortex, DL: Dorsolateral cortex prefrontal, OF: Orbitofrontal cortex, VM: Ventromedial prefrontal cortex.
